# Border-associated macrophages as gatekeepers of brain homeostasis and immunity

**DOI:** 10.1016/j.immuni.2025.04.005

**Published:** 2025-05-13

**Authors:** Mónica Vara-Pérez, Kiavash Movahedi

**Affiliations:** 1 Brain and Systems Immunology Laboratory, Brussels Center for Immunology, Vrije Universiteit Brussel, Brussels, Belgium

## Abstract

The brain’s border tissues serve as essential hubs for neuroimmune regulation and the trafficking of biomaterials to and from the brain. These complex tissues—including the meninges, perivascular spaces, choroid plexus, and circumventricular organs—balance the brain’s need for immune privilege with immune surveillance and blood-brain communication. Macrophages are integral components of these tissues, taking up key strategic positions within the brain’s circulatory system. These border-associated macrophages, or “BAMs,” are therefore emerging as pivotal for brain homeostasis and disease. BAMs perform trophic functions that help to support border homeostasis but also act as immune sentinels essential for border defense. In this review, we integrate recent findings on BAM origins, cell states, and functions, aiming to provide global insights and perspectives on the complex relationship between these macrophages and their border niche.

## Teaser

Specialized immune cells called border-associated macrophages (BAMs) guard the brain’s outer tissues, where they help maintain homeostasis and protect against disease. In this review, Vara-Pérez and Movahedi explore how BAMs develop, how they can be classified, what roles they play, and why they matter for brain health. The authors integrate recent discoveries to highlight how BAMs support and defend the brain.

## Introduction

The discovery of the blood-brain barrier (BBB) was important for establishing the concept of brain-immune privilege, which initially considered that central nervous system (CNS) antigens within the parenchyma would not reach the peripheral tissues and initiate immune responses.^1^ However, the brain cannot exist in isolation from the periphery, and the benefits of implementing a well-established degree of brain-immune surveillance are easy to postulate. Seminal work from the last decades has now provided an updated view in which intricate brain-immune interactions not only exist but are also essential for healthy brain physiology.^1^ Key locations for neuro-immune regulation are found in the brain’s border tissues, which form the interface between the parenchyma and the periphery.^2^ These include the multilayered meninges, perivascular spaces in the parenchyma, the choroid plexus (CP) within the brain’s ventricles, and the circumventricular organs (CVOs). The parenchyma and these border compartments are connected by a specialized circulatory system that allows for waste clearance and exchange of CNS antigens to accommodate immune surveillance. Macrophages, referred to as border-associated macrophages (BAMs) or CNS-associated macrophages (CAMs),^3^^,^^4^^,^^5^ are emerging as key regulators of the functionality of these brain border tissues and their fluid distribution system.

A key feature of macrophages is their profound level of tissue-specific adaptation. This was revealed following the first unbiased gene expression analyses of tissue macrophages isolated from various mouse organs.^6^ We now appreciate how tissue-specific signals within the local microenvironment critically shape the phenotype and function of macrophages. The symbiotic relationship between macrophages and their resident tissue is captured by the macrophage niche concept.^7^ The “niche” instructs the molecular identity of macrophages and provides the trophic factors required for macrophage survival and self-maintenance. The macrophage, in turn, provides a benefit for its niche. Indeed, it is now widely accepted that resident macrophages are instrumental in maintaining healthy tissue homeostasis.^8^ The tissue also acts as an important determinant of macrophage ontogeny. Across organs and tissues, macrophages can exist as short-lived cells that are continuously replaced by blood monocytes or as long-lived self-renewing populations that have an embryonic origin.^9^ This ontogenetic diversity seems to correlate with tissue accessibility. Niches that are located behind endothelial or epithelial barriers that restrict cell entry, typically contain macrophages of an embryonic origin.^9^ In sum, the nature of the tissue microenvironment shapes both the molecular identity and ontogeny of the resident macrophages. As a result, the compartmentalized brain and its heterogeneous tissue foster a diverse macrophage landscape.

## Anatomy and functions of brain border tissues

**Figure 1:**
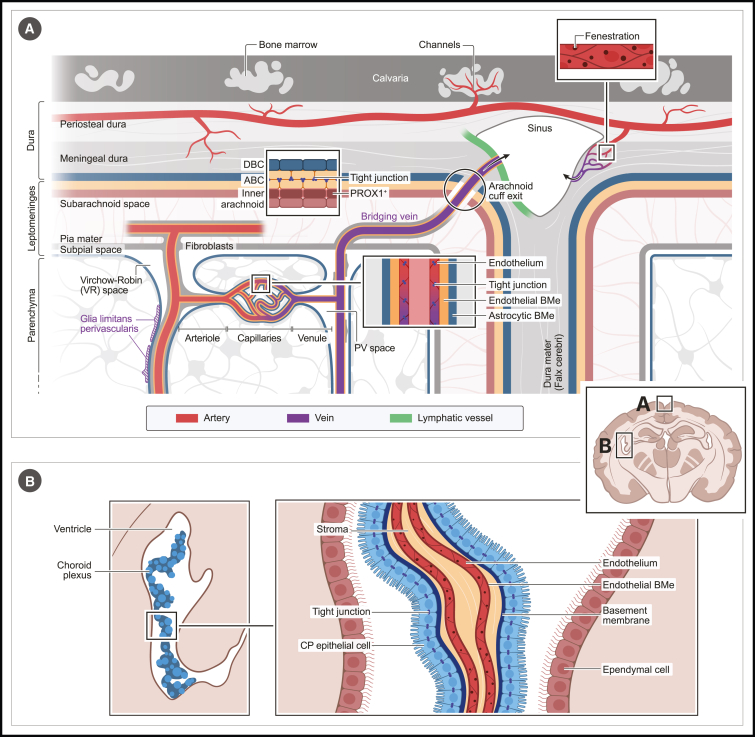
Anatomy of brain border tissues Schematic overview of brain border tissue anatomy, with (A) encompassing meninges and perivascular (PV) spaces and (B) the choroid plexus (CP). Abbreviations: dural border cell (DBC), arachnoid border cell (ABC), endothelial or astrocytic basement membrane (BMe), prospero homeobox protein 1 positive (PROX1+) cells.

To elucidate the biology of BAMs and their role in brain physiology, it is essential to understand their niche. We will, therefore, begin by exploring the unique characteristics of the brain's border tissues (Figure 1), detailing how these components integrate into a complex system responsible for regulating brain fluid dynamics, facilitating the trafficking of biomolecules and cells, and maintaining immune surveillance.

The brain’s outer surface is covered by the meninges, a highly complex three-layered structure consisting of the dura mater as the outermost layer, followed by the arachnoid and pia mater, which together are referred to as the leptomeninges (Figure 1A). The dura mater consists of thick connective tissue, composed of fibroblasts and a dense extracellular matrix containing various collagen species.^10^^,^^11^^,^^12^^,^^13^ The dura mater segregates into a periosteal layer, which is attached to the calvarium, and a meningeal layer. Of note, both of these layers remain attached to the calvaria during dissection. The periosteal and meningeal layers separate at the level of the sinuses, which are large channels that drain venous blood from the brain.^14^ At these points, the meningeal dura folds inward, forming the dural reflections, such as the falx cerebri that separates the cerebral hemispheres (Figure 1A). Sinuses, composed of dura mater lined with endothelium, lack tunica media and valves.^14^ These structural features, combined with their large luminal area, result in a relatively slow blood flow in the sinuses. This could render sinuses more susceptible to blood-borne pathogens, which may explain why they harbor many immune cells and represent an important neuroimmune interface.^15^ From an immune perspective, the dura mater is also often divided into sinus and non-sinus regions. Running parallel along the dural sinuses are also lymphatic vessels, which drain fluid to the deep cervical lymph nodes.^16^^,^^17^ The dura mater is also richly innervated by nerve fibers,^13^ including nociceptors that are implicated in local neuroimmune signaling.^18^

Meningeal arteries supply oxygen-rich blood to the dura,^13^ which branch into capillaries that lack tight junctions and have fenestrations,^19^ facilitating the exchange of blood-derived molecules and immune cells. Dural vessels also connect to bone marrow (BM) cavities within the calvaria through ossified channels^20^^,^^21^ (Figure 1A). These vascular connections enable immune cells to migrate directly from the skull marrow to the dura, bypassing the need for blood trafficking.^22^^,^^23^ It has also been suggested that factors produced within the brain diffuse through the dura-skull channels and locally modulate BM hematopoiesis and cell egress.^24^ The skull marrow does have unique features, including life-long expansion, greater resilience to aging, and distinct transcriptional states as compared with the marrow in other bones.^25^^,^^26^

The innermost layer of the dura consists of dural border cells (DBCs), which are elongated, flattened fibroblasts^10^^,^^11^^,^^12^^,^^19^ (Figure 1A). DBCs have irregular patterns and thereby form many extracellular spaces and are connected to the underlying layer of arachnoid barrier cells (ABCs). ABCs form two layers of cells that lack extracellular spaces and are interconnected by tight junctions, forming an essential barrier between the blood-accessible dura and the underlying subarachnoid space (SAS).^10^^,^^19^ Historically, it was believed that a subdural space existed between the DBC and ABC layers. However, this is not a pre-existing space but one that can be formed upon trauma, which can lead to an accumulation of fluids within the structurally weak DBC layer, resulting in a subdural hematoma.^10^

Beneath the ABCs are extra layers of fibroblasts,^27^ including a PROX1^+^ subset, which was recently proposed to delineate an additional compartment within the SAS,^28^ although under normal conditions, PROX1^+^ fibroblasts are attached to the ABCs.^27^^,^^29^^,^^30^ Fibroblasts within this inner arachnoid layer also form trabeculae, which bridge the SAS and attach to the pia mater.^10^^,^^11^ Of note, although common in humans, trabeculae are absent in the mouse cortex but can be found in the mouse spinal cord.^29^ The pia mater is composed of flattened fibroblasts connected by adherens junctions but lacking tight junctions.^10^^,^^11^^,^^12^^,^^19^ Beneath the pia mater lies the glia limitans superficialis, a layer of specialized astrocytes that line the brain parenchyma and produce a basement membrane.^31^^,^^32^ A subpial space separates the pia mater from the glia limitans.

The internal carotid and vertebral arteries supply the brain with oxygenated blood.^32^ These branch and traverse the SAS, where they are covered by pial fibroblasts and anchored by trabeculae.^33^ Blood vessels within the SAS contain tight junctions, forming the blood-leptomeningeal barrier (BLMB).^34^ The BLMB differs from the BBB in that its endothelial cells are not covered by astrocytic endfeet. Additionally, unlike the BBB, the BLMB forms a diffusion barrier between peripheral blood and the cerebrospinal fluid (CSF) flowing within the SAS. However, the BLMB is distinct from the blood-CSF barrier (BCSFB), which is defined as the epithelial diffusion barrier situated at the CP. Veins within the SAS connect to the dural sinuses via so-called bridging veins, which traverse the ABC layer. Recent work has shown that discontinuities in the ABC layer exist around bridging veins, forming so-called arachnoid cuff exits (ACEs) (Figure 1A), which may allow for direct communication between the SAS and dural compartments, including for the passage of immune cells.^30^ The arteries and veins within the SAS vertically penetrate the parenchyma and thereby create perivascular spaces, which are filled with interstitial fluid (ISF).^35^ Perivascular spaces are delimited by the basement membranes of vascular endothelial cells and the glia limitans perivascularis, a barrier-like layer formed by astrocytic endfeet. Although these two basement membranes form perivascular spaces around the penetrating arteries, arterioles, venules, and veins, they are fused around brain capillaries.^35^ Penetrating arteries and their arterioles are also covered by pial fibroblasts, but these are lost as they branch into capillaries and few fibroblasts are seen around venules and veins^33^ (Figure 1A). The large perivascular spaces around penetrating arteries and veins are also specifically referred to as Virchow-Robin (VR) spaces.^35^ Whereas the pia mater separates the SAS and perivascular compartments, the subpial space is continuous with the perivascular space^33^^,^^36^ (Figure 1A). However, pores or stomata within the fibroblast lining of leptomeningeal vessels and pia allow for fluid exchange between the SAS and subpial/perivascular space.^37^

Vessels within the brain parenchyma contain tight junctions. Additionally, the presence of the glia limitans, two layers of basement membrane, pericytes, and capillary-associated microglia, together form a neurovascular unit that constitutes the BBB.^32^ The BBB is a highly restrictive barrier, making the brain parenchyma the most immune-privileged site within the brain. However, the brain and its neural circuits require the ability to sense blood-derived signals or relay brain information to the periphery through the secretion of neuropeptides. This is accomplished by specialized structures called CVOs, which can be found within the parenchyma associated with the third and fourth ventricles.^38^ To enable blood-brain communication, capillaries within the core of the CVOs lack tight junctions, are devoid of glia limitans, and exhibit fenestrations.^38^ CVOs are therefore accessible to blood-derived molecules and can be considered parenchymal border tissues. In most CVOs, capillaries are bordered by two basement membranes that delineate large perivascular spaces, which are contacted by neuronal processes.^38^ A special type of ependymal cells called tanycytes, which lack cilia and are interconnected by tight junctions, form a barrier between the CVO parenchyma and the CSF within the brain ventricles.^39^^,^^40^^,^^41^ Tanycytes and perhaps other cell types may also form a barrier between the CVO and the adjacent parenchyma, although this remains incompletely understood.

The brain has four interconnected ventricles that are filled with CSF and lined by ciliated ependymal cells.^42^ CSF is primarily produced by the CP, which floats within the ventricles^42^^,^^43^^,^^44^ (Figure 1B). The CP consists of a single layer of cuboidal epithelial cells, which are related to ependymal cells. However, CP epithelial cells contain microvilli instead of cilia and secrete a basement membrane at their basal side.^42^ CP epithelial cells enclose a loose connective tissue referred to as the CP stroma (Figure 1B). The stroma is highly vascularized and contains fenestrated capillaries, lacking tight junctions. However, access to ventricular CSF is restricted by the CP epithelial cells, which are interconnected by tight junctions and form the BCSFB.^42^^,^^43^^,^^45^ The CP develops from the tela choroidea, a vascularized connective tissue layer considered as ependymal cells attached to the pia mater. As a result, the CP stroma is in continuity with the subpial space and therefore also with the perivascular spaces.^42^ Remarkably, ABCs or ABC-like cells expressing tight junction proteins such as CLAUDIN-11 seem to extend into the tela choroidea, likely forming a diffusion barrier between the blood-accessible CP stroma and the perivascular spaces and SAS.^12^^,^^46^

CSF within the lateral ventricle may move to the third ventricle via the foramen of Monro and pass through the cerebral aqueduct to reach the fourth ventricle. From there, CSF reaches the cranial SAS by passing the lateral or median aperture.^44^ The latter connects to the cisterna magna, a large SAS region near the cerebellum often used for drug or tracer injection studies in rodents. There is clear evidence that CSF flowing within the SAS mixes with the ISF of the perivascular spaces.^37^^,^^47^ The glymphatic model proposes a bulk CSF flow, moving into perivascular spaces around arteries in the direction of the blood flow, perhaps driven by peristaltic pumping by arterial pulsations.^47^^,^^48^ CSF would subsequently circulate across the parenchyma and intermix with ISF, followed by a perivenous efflux.^48^ This would allow for efficient waste clearance and exchange of CNS antigens. However, there is still considerable debate on whether CSF/ISF exchange primarily occurs via convective flow or diffusion.^37^^,^^47^

Finally, CSF within the SAS needs to be reabsorbed toward the periphery. Historically, it was assumed that most CSF directly drains into the dural sinuses via structures called arachnoid granulations (AGs).^44^^,^^49^ AGs are protrusions of arachnoid mater into the dural sinuses. However, AGs are not observed in human neonates and some adults and are absent in mice.^50^ Furthermore, the notion that most CSF is removed via the bloodstream is now challenged, as a growing body of evidence has indicated that CSF is efficiently removed via lymphatic drainage, which would allow for immune surveillance of CSF antigens in lymph nodes.^16^^,^^17^^,^^51^^,^^52^^,^^53^^,^^54^^,^^55^^,^^56^ Dural lymphatic vessels are found both in the dorsal and basal parts of the brain.^16^^,^^17^^,^^54^^,^^56^ However, CSF within the SAS needs to pass the ABC layer to reach the lymphatic vessels. Recent work suggests that this is facilitated by ACEs around bridging veins, allowing CSF to reach the dorsal dural lymphatics.^30^ In humans, this may also occur via AGs that, instead of connecting to the sinus, could also offer a conduit to the adjacent dural tissue.^57^ Another important exit route for CSF is along cranial and spinal nerves. Within the brain, this is thought to primarily occur via the olfactory bulb-cribriform axis.^52^^,^^58^^,^^59^ Sensory neurons in the olfactory epithelium project large axon bundles through the cribriform plate toward the olfactory bulb. Lymphatic vessels and CSF travel along these axonal tracts, allowing CSF drainage via the nasopharyngeal lymphatic plexus.^52^^,^^56^^,^^58^^,^^59^

## Macrophages in brain border tissues

Macrophages are the predominant immune cell type within all border tissues of the brain. BAMs are embedded within the connective tissue of the dura mater and CP stroma, where they have access to blood-derived molecules but are shielded from the CSF by ABCs and CP epithelial cells, respectively. However, tracer injections have suggested that dural BAMs do have access to CSF-derived antigens.^15^ Macrophages also reside in the leptomeninges, where electron microscopy has revealed that they are either attached to the pia mater or located within the subpial space.^60^^,^^61^ In perivascular spaces, macrophages are located between the two basement membranes and are predominantly found around αSMA^+^ arteries and arterioles, where they interact with arterial vascular smooth muscle cells (VSMCs).^62^^,^^63^ Leptomeningeal and perivascular BAMs, also collectively called parenchymal border macrophages,^62^ are shielded from blood-borne products through the BLMB but have access to ISF and CSF. Indeed, leptomeningeal and perivascular BAMs efficiently sample tracers injected in the CSF or parenchyma.^62^^,^^64^ These macrophages are locally confined and do not enter the brain parenchyma to clear cellular debris.^65^ Macrophages can also be found in the ventricles, lining the apical surface of the CP epithelium. These macrophages are known as Kolmer’s epiplexus cells or epiplexus BAMs.^66^ Related to these are intraventricular macrophages that are found on the ependymal surface of the ventricles, although these cells are rare during homeostasis.^66^ Epiplexus BAMs and intraventricular macrophages are not embedded in tissue but bathe in the CSF. Finally, within the CVOs, macrophages can be found in perivascular spaces that—in contrast to normal brain parenchyma—exist around capillaries.^38^ Thus, many CVO BAMs are capillary-associated perivascular macrophages with access to blood-derived products. These macrophages have been proposed to act as a barrier, restricting blood-borne macromolecules from reaching the parenchyma through rapid phagocytosis.^67^

Microglia and BAMs strictly depend on CSF1R signaling for their development and survival. CSF1R receptor deficiency results in a complete loss of microglia and BAMs.^68^^,^^69^ These cells are also highly sensitive to CSF1R inhibition by small-molecule inhibitors such as PLX3397, which can induce a rapid depletion of brain macrophages,^70^ including all types of BAMs.^5^ CSF1R ligands include CSF1 and interleukin (IL)-34, which can show overlapping expression in border tissues, such as the leptomeninges.^71^ In peripheral tissues, macrophages rely on CSF1R ligands produced by specific niche cells, and loss of expression within these cells impairs their survival. Examples include CSF1 produced by red pulp fibroblasts that maintain red pulp macrophages in the spleen^72^ or non-classical monocytes that specifically need to scavenge CSF1 produced by endothelial cells to survive.^73^ Furthermore, niche cells not only provide the essential CSF1R ligands but also help imprint the identity of the tissue macrophages.^74^ Also within the brain border tissues, niche cells are starting to be discovered. In the perivascular spaces, arterial VSMCs are crucial for maintaining perivascular BAMs.^63^ Other likely niche cells and essential sources of CSF1/IL-34 are likely to be subpopulations of meningeal and CP fibroblasts and endothelial cells,^75^ although this still needs to be determined.

## BAM subsets and cell states in mice and humans

The nature of the tissue environment has a profound impact on the brain macrophage phenotype. Notably, microglia and BAMs exhibit distinctively different identities, evident at morphological, transcriptional, and functional levels. Microglia are characterized by their ramified morphology, whereas BAMs exist in elongated or amoeboid forms.^3^^,^^5^ At the transcriptional level, microglia and BAMs are highly distinct. Many of the key signature genes that define mouse microglia, identified in early bulk gene expression studies,^76^^,^^77^ are not or poorly expressed in BAMs, including *P2ry12*, *Tmem119*, and *Sall1*.^3^^,^^5^ Conversely, genes such as *Ms4a7*, *Cybb*, and *Tgfbi* are universally high in BAMs but absent in homeostatic microglia. At the protein level, BAMs can be distinguished from homeostatic microglia by their higher expression of CD206, F4/80, and CLEC12A.^4^^,^^5^ However, it is important to note that BM-derived monocytes that can engraft the brain parenchyma as microglia-like cells under specific conditions, display a hybrid microglia-BAM identity.^78^^,^^79^^,^^80^^,^^81^ These cells lack certain microglial genes such as *Sall1*, while expressing many BAM-related markers, including *Ms4a7*, *Cybb*, *Tgfbi*, and *Mrc1/*CD206. Therefore, transcriptional differences between microglia and BAMs are partly shaped by ontogeny. This relates to the inability of adult BM-derived microglia to turn on *Sall1* expression, which is essential for instructing a core component of the microglial transcriptome.^82^

The transcription factors and gene networks that determine whether brain macrophages adopt a microglial or BAM identity are gradually being elucidated. IRF8 is essential for establishing the proper microglial transcriptional program.^5^^,^^83^ In *Irf8* knockout (KO) mice, microglia lose key aspects of their identity and express many markers typically associated with BAMs, such as *Ms4a7*, *Tgfbi*, *Pf4*, and *Clec4n*.^5^^,^^83^ Many of these dysregulated genes are likely a consequence of the loss of SALL1,^82^ for which IRF8 acts as an upstream regulator.^5^^,^^83^ SALL1 expression also depends on SMAD transcription factors.^84^ Transforming growth factor β (TGF-β) signaling induces phosphorylation of SMAD2 and SMAD3, which then associate with SMAD4 and translocate to the nucleus.^85^ As a result, TGF-β is essential for instructing the microglial identity,^76^^,^^86^ and loss of SMAD4 in microglia results in the induction of BAM signature genes.^87^ Conversely, TGF-β and SMAD4 are not required for establishing BAM identity.^86^^,^^87^ Thus, IRF8, TGF-β, and SMADs collectively drive the expression of SALL1, which imprints a core aspect of the microglial identity distinct from that of BAMs.

**Figure 2:**
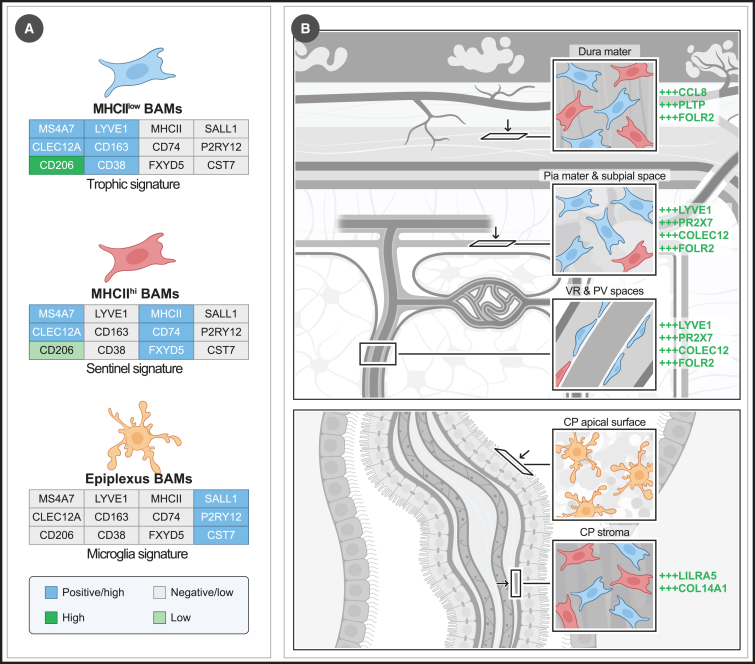
BAM subsets and their distribution throughout the brain (A) Overview of main border macrophage identities or cell states, with key defining markers. (B) Distribution of BAMs across border tissues. Examples of protein markers enriched in MHCII^low^ BAMs from specific border regions are shown in green. Abbreviations: Virchow Robin (VR), perivascular (PV), and choroid plexus (CP).

Importantly, BAMs exist in two primary states. One state is characterized by high expression of major histocompatibility complex (MHC) class II (MHCII) molecules, whereas the other subset is MHCII-negative to low but exhibits higher levels of CD38, LYVE1, and CD163^4^^,^^5^ (Figure 2A). Transcriptional profiling reveals numerous genes that are differentially expressed between these states. Many genes enriched in MHCII^low^ BAMs encode scavenger and phagocytic receptors (e.g., *Cd36*, *Msr1*, *Cd163*, *Stab1*, *Clec4n*, and *Clec10a*) or tissue-trophic factors (e.g., *F13a1* and *Igf1*),^5^^,^^62^^,^^88^ suggesting tissue-supportive functions through apoptotic cell clearance, lipid and hemoglobin scavenging, and tissue repair. In contrast, MHCII^hi^ BAMs express, in addition to MHCII, other genes typically associated with antigen-presenting cells, such as *Cd74* or *Fxyd5*, highlighting an immune sentinel signature. Although in adults both states are present in all border tissues, MHCII^hi^ BAMs are primarily found in the dura mater and CP stroma^5^ (Figure 2A), regions containing fenestrated blood vessels that may be more exposed to blood-borne pathogens. Within the leptomeninges and perivascular spaces, MHCII^hi^ BAMs comprise a minority.^5^^,^^62^ The phenotype of BAMs within the CVOs has not been described in detail yet.

Although BAMs can be classified based on these two states, it does not mean that these states are identical across all border tissues or that additional heterogeneity may not exist. MHCII^low^ BAMs in particular exhibit clear tissue-specific adaptations.^5^ For example, although leptomeningeal and perivascular MHCII^low^ BAMs show uniform high expression of LYVE1, not all MHCII^low^ BAMs in the dura mater express LYVE1, whereas CP BAMs are mostly LYVE1 negative.^5^ Additional examples of border-specific gene signatures, such as the absence of FOLR2 in CP BAMs, are noted in Figure 2. In the perivascular spaces, Siret et al. also reported the existence of a LYVE1^+^ CX3CR1^−^ subset of BAMs.^89^ However, this was based on heterozygous *Cx3cr1*-GFP mice as a proxy for CX3CR1 expression, without protein-level confirmation. Given the potential for monoallelic expression at the *Cx3cr1* locus, as observed for certain genes,^90^ or allele-specific silencing of GFP, confirming the presence of CX3CR1^−^ perivascular BAMs in wild-type (WT) mice at the protein level is important.

One subset of border macrophages that stands out from all others is found in the CP. Fate mapping using *Sall1*^CreER^ mice revealed the presence of *Sall1*^+^ macrophages on the apical surface of the CP epithelium.^5^ Similarly, epiplexus BAMs were also observed using the *P2ry12*^CreER^ microglia fate mapper.^91^ Remarkably, these SALL1^+^P2RY12^+^ epiplexus macrophages do not express typical BAM markers, such as *Ms4a7* or *Cybb*, and do not adhere to the MHCII^low^ or MHCII^hi^ states as they are CD206^−^LYVE1^−^MHCII^−^^5^ (Figure 2A). Via single-cell RNA sequencing (scRNA-seq), we have shown that these SALL1^+^ epiplexus BAMs closely resemble microglia but exhibit a non-homeostatic transcriptional state, comparable—but not identical—to those of disease-associated, proliferative-region-associated, or white-matter-associated microglia.^5^^,^^92^^,^^93^^,^^94^ The microglia-like state of these cells has also been confirmed in a separate study that integrated brain macrophages from multiple independent datasets.^95^ SALL1 expression is not observed in adult BM-derived brain macrophages, including BM-derived microglia,^78^^,^^79^^,^^80^^,^^81^ thus suggesting that SALL1^+^ epiplexus cells share an origin with embryonic microglia. It remains unclear whether all epiplexus macrophages consist of microglia-like cells or whether an additional SALL1^−^ epiplexus population that expresses conventional BAM markers also exists. The numbers of SALL1^+^ epiplexus BAMs are low in homeostatic brains, where most CP macrophages are stromal BAMs. However, during *Trypanosoma brucei* infection of the brain, in which parasites accumulate in the CP, a large increase of SALL1^+^ epiplexus BAMs is observed,^96^ indicative of a highly dynamic population. However, during acute inflammation, such as shortly after intraventricular LPS injection, SALL1^−^ CSF-derived epiplexus BAMs can also be observed.^97^

The identities of human BAMs have recently started to emerge. Independent studies have profiled human immune cells from the leptomeninges, dura mater, CP, and parenchymal tissue via scRNA-seq.^98^^,^^99^^,^^100^ This has revealed BAMs with a high expression of *MRC1*, *LYVE1*, *CD163*, and *F13A1*,^98^^,^^99^ a gene signature that closely mirrors that of mouse MHCII^low^ BAMs. These were the dominant BAMs in the leptomeninges,^98^ whereas this signature was less obvious in the human dura mater in one study.^100^ Interestingly, Sankwoski et al. also observed putative CP epiplexus BAMs that co-clustered with activated microglia.^99^ Whether a human counterpart to MHCII^hi^ BAMs exists was not immediately evident from these studies, although variations in HLA-DR (human MHCII) expression were observed among human BAMs across different border regions.^99^ In our recent work, we intracerebrally transplanted human blood monocytes in neonatal mice expressing human CSF1 on a *Rag2/Il2rg*-deficient background.^81^ This resulted in the engraftment of transplanted monocytes as human BAMs in the border tissues of the mouse brain. scRNA-seq revealed the presence of human MRC1^hi^ LYVE1^hi^ CD163^hi^ F13A1^hi^ macrophages, closely mirroring the reported phenotype of BAMs from human brains. Additionally, we identified a subset with low expression of these genes but with an enriched expression of *HLA-DRA*, *GPNMB*, *APOC1*, *APOE*, and *ITGAX*. Whether these BAMs represent MHCII^hi^ BAMs that can be found in human brains remains to be confirmed.

## Origins and states: From embryo to birth

Macrophages colonize the mouse brain as early as E9.5 of gestation.^101^ A divergence between microglial and BAM identities begins shortly thereafter. Although most brain macrophages at E10.5 are CD206^+^, the expression of this marker is gradually lost in parenchymal macrophages, while being maintained in the mesenchyme.^86^^,^^102^ This is a result of cell state transitions, with CD206^+^ cells converting into CD206^−^ microglia.^63^^,^^103^ Interestingly, around E12.5, CD206^+^ macrophages from the mesenchyme still actively infiltrate the brain parenchyma, moving from the ventricles into the pallium, where they subsequently undergo reprogramming and become CD206^−^ microglia.^103^ Loss of CD206 expression and adoption of a microglial morphology is from E12.5 onward already dependent on SALL1.^102^ By E14.5, the parenchyma is mostly populated by CD206^−^P2RY12^+^ cells, whereas CD206^+^LYVE1^+^ macrophages that transcriptionally resemble BAMs are found in the embryonic meninges and CP stroma.^63^^,^^86^^,^^102^ From E14.5 onward, Utz et al. also observed CD206^+^LYVE1^+^ macrophages surrounding some parenchymal vessels.^86^ However, Masuda et al.^63^ reported that perivascular spaces develop postnatally, with perivascular BAMs appearing only after P7 and deriving from leptomeningeal BAMs. Supporting this, recent work has shown that perivascular fibroblasts also emerge around P7 and, like perivascular BAMs, derive from a meningeal source.^104^

Where do the CD206^+^ BAMs and CD206^−^ microglia cells that colonize the embryonic brain originate from? Fate mapping using the *Cdh5*^CreER^ to label the yolk sac hemogenic endothelium revealed that both populations derive from yolk sac erythro-myeloid progenitors (EMPs) and not from definitive hematopoiesis.^86^ Furthermore, tamoxifen administration at E7 in *Runx1*^CreER^ mice suggested that, like microglia, BAMs in the E18.5 brain are derived from primitive EMPs.^86^ However, an intriguing, but unexplained, observation by Utz et al. was that tamoxifen tagging at E9 in *Cx3cr1*^CreER^ mice initially labels all brain macrophages to an equal extent, whereas from E14 onward the percentage of labeled BAMs drops significantly in the meninges and CP while that of microglia remains stable.^86^ This suggests that another unlabeled progenitor, perhaps late EMP progeny, is supplementing BAMs in these compartments.

Although there is information on when ventricular macrophages are observed throughout development,^66^ it is currently not clear when SALL1^+^ cells first appear on the CP epithelium and through which route they arise. The CP stroma develops from cephalic mesenchyme, which also gives rise to meningeal tissue. Thus, epiplexus BAMs may derive directly from CD206^+^ CP stromal macrophages that migrate between CP epithelial cells. A second possibility is that CD206^+^ macrophages migrate from the mesenchyme into the ventricles, for example, by crossing the roof plate,^103^ and move onto the CP epithelial surface. In both scenarios, CD206^+^ macrophages would need to be reprogrammed locally toward a CD206^−^ SALL1^+^ state. A third possibility is that SALL1^+^ epiplexus BAMs originate from parenchymal microglia that migrated to the CP via the ventricles. Although two-photon *in vivo* imaging has shown that at E12.5 most intraventricular macrophages migrate from the ventricle into the parenchyma, the authors also occasionally observed microglial migration in the reverse direction.^103^ *Ex vivo* imaging of E11 slices has also demonstrated this microglial behavior.^105^ As the ependymal layer in the ventricles is continuous with the CP epithelium,^42^ this would offer ventricular microglia a direct path toward the CP epithelium. If the parenchyma-to-CP route is correct, imprinting of the SALL1^+^ microglial identity might occur within the parenchyma rather than locally on the CP epithelium. It also remains unclear whether SALL1^+^ epiplexus BAMs in adults self-renew locally or whether a parenchyma-to-CP migration route remains active. Alternatively, parenchymal microglia may only serve as an emergency supply during inflammation and disease, specifically for conditions that require the expansion of SALL1^+^ epiplexus BAMs.^96^

## Origins and states: From birth to old age

BAMs display low heterogeneity during development and at birth, exhibiting homogeneous expression of markers such as LYVE1 and CD163 while lacking MHCII expression.^5^^,^^75^^,^^86^^,^^106^^,^^107^^,^^108^ At this stage, MHCII^low^ BAMs from different border regions also exhibit greater similarity. This is particularly evident for CP BAMs, which initially more closely resemble meningeal MHCII^low^ BAMs, displaying higher expression levels of CD206, *Clec4n*, and *Slc40a1*.^5^^,^^75^^,^^93^ The transcription factor IRF8 plays an important role in shaping the adult CP BAM identity.^5^ Its deletion results in CP BAMs retaining a more meningeal-like MHCII^low^ BAM phenotype, characterized by a sustained expression of *Clec4n*, *Slc40a1*, and *Lyve1*.^5^

MHCII^low^ BAMs in the CP and dura mater are slowly replaced by monocytes throughout adult life, whereas MHCII^low^ BAMs in the leptomeninges and perivascular spaces remain of embryonic origin.^5^^,^^109^ It is currently unclear what factors determine the enhanced monocyte engraftment observed in the dura mater and CP. It has been hypothesized that monocyte engraftment is stimulated by low-grade inflammation,^110^ which may be more prominent in the dura and CP due to their increased exposure to blood-derived products. Alternatively, BAMs in the dura and CP may be less efficient in self-renewal due to differences in the local tissue niche. In peripheral barrier tissues, such as the skin and intestine, increased macrophage turnover and monocyte engraftment are commonly observed.^111^ As many intracellular pathogens infect macrophages, frequent replacement of tissue macrophages may help to eliminate pathogen reservoirs.^110^ This is also relevant for the dura and CP, which can act as entry sites for pathogens.^18^^,^^96^^,^^106^^,^^107^

**Figure 3:**
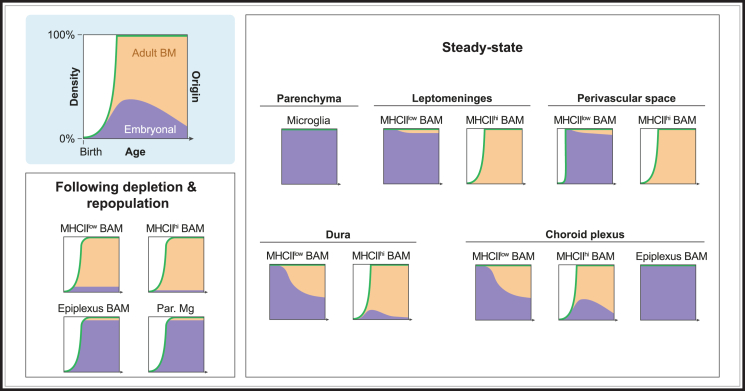
Overview of the origin and developmental kinetics of the indicated brain macrophage populations within the various brain compartments The situation is shown under normal healthy steady-state conditions (steady state) and following depletion and repopulation of brain macrophages using CSF1R inhibitors (depletion and repopulation).

MHCII^hi^ BAMs first appear shortly after weaning, at around 3 weeks of age.^5^ They initially arise through the reprogramming of embryonic BAMs as well as from newly recruited monocyte-derived cells.^5^ MHCII^hi^ BAMs become increasingly prominent as mice age, in all border regions.^4^^,^^5^^,^^62^^,^^75^^,^^95^^,^^106^^,^^107^^,^^108^^,^^112^ Remarkably, MHCII^hi^ BAMs in the leptomeninges and perivascular spaces appear to be completely monocyte derived.^81^^,^^87^ Therefore, all border regions contain BAMs of both embryonal and postnatal BM origins, which develop with distinctive kinetics, as summarized in Figure 3. This is in clear contrast to the parenchyma, where microglia are exclusively embryonically derived. Monocytes that replace BAMs may be blood-derived but can potentially also originate directly from the skull BM by migrating through ossified channels (Figure 1A).^23^ The signals and microenvironmental changes that drive the appearance and progressive accumulation of MHCII^hi^ BAMs remain incompletely understood. There is evidence that this is positively correlated with exposure to microbiota, as germ-free animals harbor 10%–20% fewer MHCII^hi^ BAMs as compared with age-matched controls from conventional or SPF facilities.^5^^,^^113^

Even though under healthy steady-state conditions, MHCII^low^ BAMs in the leptomeninges and perivascular spaces maintain their EMP origin throughout life, recent work has shown that this can easily be lost. Following PLX3397-induced depletion of brain macrophages, embryonically derived MHCII^low^ BAMs in the leptomeninges and perivascular spaces are replaced by monocyte-derived counterparts (Figure 3).^81^^,^^114^ This is in stark contrast to microglia, which even after widespread depletion can rapidly repopulate from a small number of surviving cells following PLX3397 withdrawal.^81^^,^^115^^,^^116^ Why can microglia repopulate with minimal monocyte engraftment whereas leptomeningeal and perivascular BAMs are replaced, despite both being behind a brain barrier and sharing the same embryonic precursor? We hypothesize that this difference reflects the lower self-renewal capacity of BAMs compared with microglia, likely due to niche-imprinting.^81^ Consequently, PLX3397-induced depletion will allow for monocyte engraftment in these border regions. Importantly, replacement of leptomeningeal BAMs is not only a consequence of PLX3397 treatment but is also observed following injury or disease,^114^ highlighting the susceptibility of embryonic BAMs to replacement after general macrophage loss. This tendency is commonly observed in many tissue macrophages,^7^ except for microglia. Remarkably, monocyte-derived MHCII^low^ BAMs that engraft the leptomeninges and perivascular spaces following PLX3397-induced depletion are transcriptionally distinct from their original embryonic counterparts.^81^ This indicates that ontogeny shapes the transcriptional identity of MHCII^low^ BAMs within these border regions, as it also does for microglia. However, the identities or cell states of brain macrophages may also be shaped by the time of residency within the tissue. It remains to be assessed whether ontogeny-related differences in BAMs may resolve or “erode” over extended periods of tissue residency, as has been suggested for monocyte-derived microglia.^117^

## BAMs regulate tissue and vascular homeostasis

Through their expression of scavenging and phagocytosis-related receptors, BAMs are expected to play important roles in waste clearance within the brain. This function is particularly relevant for perivascular and leptomeningeal BAMs, given their strategic positions within the glymphatic system. Particle injection into the cisterna magna has demonstrated that MHCII^low^ BAMs, rather than MHCII^hi^ BAMs, are primarily responsible for particle uptake.^62^ These parenchymal border macrophages also phagocytose erythrocytes upon subarachnoid hemorrhage. For example, BAM depletion prior to prechiasmatic cistern injection of erythrocytes results in the appearance of blood clots on the ventral surface of the brain.^118^ Erythrocyte clearance is likely performed by BAMs expressing receptors for erythrocyte uptake, such as CD36 and STABILIN-1,^119^^,^^120^ in addition to CD163, which is involved in hemoglobin clearance.^121^

Gene ontology and pathway analysis of genes enriched in MHCII^low^ BAMs have suggested roles in vascular regulation and lipid metabolism.^5^ A high-fat diet induces the upregulation of VEGFA in perivascular BAMs, which may help to restore cerebral glucose metabolism and prevent cognitive decline.^122^ The expression of the ferritin receptor SLC40A1 in BAMs located close to border vessels suggests a role in local iron homeostasis.^75^

Related to their involvement in scavenging and vascular homeostasis, BAMs have been shown to regulate CSF flow dynamics.^62^ MHCII^low^ BAMs in the leptomeninges and perivascular spaces control extracellular matrix remodeling via the expression of metalloproteases and matrix remodeling factors. This prevents excessive collagen and laminin accumulation around vessels, which would otherwise constrict the perivascular space and disrupt CSF flow.^62^ This intriguing connection between brain macrophages and CSF regulation extends further. CSF1R-deficient mice and rats, which lack brain macrophages, also develop hydrocephalus.^68^^,^^69^ The underlying mechanism remains unclear. One possibility is that the absence of BAMs disrupts debris clearance within the ventricular system, leading to a blockage. Alternatively, the lack of brain macrophages might impair lymphatic CSF drainage or compromise the functionality of CP epithelial cells.

Whether MHCII^low^ and epiplexus BAMs have overlapping or complementary roles remains to be clarified. Given their localization, epiplexus BAMs may be essential for scavenging particles within the ventricles, as shown by tracer studies,^123^ or could rapidly phagocytose material that passes the BCSFB.^124^ For example, an electron microscopy study revealed that intravenously injected horseradish peroxidase (HRP) extravasates from fenestrated capillaries into the CP stroma, where it is endocytosed by CP epithelial cells, which subsequently release it into the CSF via exocytosis. As a result, epiplexus BAMs progressively accumulate HRP, showing extensive accumulation in lysosomes.^124^ Epiplexus BAMs may also play a role in regulating CP epithelial function. Following brain infections or hemorrhage, cytokines secreted by epiplexus BAMs may induce excessive CSF production by CP epithelial cells, thereby triggering hydrocephalus.^66^^,^^125^ Epiplexus BAMs can also help repair the CP epithelial barrier following inflammation.^97^

Brain-related diseases often involve a strong immunological component, which has been directly associated with BAMs, particularly in the context of neurovascular dysfunction. Hypertension (or high blood pressure) has been shown to alter the BBB^126^ and is considered a risk factor for stroke and Alzheimer’s disease (AD), among other pathologies.^126^ In mouse models of chronic angiotensin II (ANGII)-induced hypertension, perivascular BAMs sense ANGII that diffuses into the perivascular spaces, resulting in the production of reactive oxygen species (ROS) via the enzyme NOX2, thereby contributing to pathology.^126^^,^^127^ In a model of salt-sensitive hypertension, an increase of IL-17 in the CSF, possibly originating from dural T cells, was also shown to act on perivascular BAMs and induce dysfunction, again likely via NOX2-mediated ROS production.^128^ A deleterious effect of ROS produced by perivascular BAMs on neurovascular dysfunction has been reported in knockin mice carrying the human apolipoprotein E4 variant, a major genetic risk factor for AD that also contributes to microvascular pathology.^129^ In line with their role in vascular dysfunction, emerging evidence implicates BAMs in cerebral amyloid angiopathy (CAA), a condition characterized by the deposition of amyloid-β (Aβ) in cerebral blood vessels.^130^ CAA can occur as a feature of AD but also develops independently and may be partly driven by impaired vascular Aβ clearance.^130^ Although some studies suggest that BAMs contribute to CAA through excessive perivascular ROS production,^131^^,^^132^ others have shown that perivascular and leptomeningeal BAMs facilitate Aβ clearance^62^ and that their depletion exacerbates CAA.^133^ The role of BAMs in AD extends beyond neurovascular functions. AD is a chronic neurodegenerative disorder characterized by the accumulation of extracellular Aβ plaques, which triggers the spread of intracellular neurofibrillary tangles composed of hyperphosphorylated tau protein within neurons.^134^ In addition, excessive synaptic pruning, particularly by activated microglia, contributes to synapse loss and cognitive decline.^135^ Perivascular BAMs have been reported to influence this process by producing SPP1/osteopontin, which promotes excessive synaptic pruning by microglia.^136^ However, the involvement of BAMs in AD pathology is complex, as their depletion has also been shown to exacerbate tau pathology.^137^

In conclusion, although BAMs play important supportive roles during homeostasis, dysregulation of their functions in chronic disease contexts may contribute to pathology and neurodegeneration.

## BAMs lead brain border defense against pathogens and may bridge adaptive immunity during neuroinflammation

Recent research highlights BAMs as crucial protectors against invading pathogens. Upon viral infection, BAM depletion leads to fatal lymphocytic choriomeningitis virus (LCMV)-driven meningitis.^107^ To determine whether this protective role is primarily linked to MHCII^low^ or MHCII^hi^ BAMs, Rebejac et al. developed experimental strategies to manipulate the ratio of these subsets in the dura mater. Interestingly, reducing the proportion of MHCII^hi^ BAMs significantly increased the LCMV viral load within the first 48 h post infection.^107^ These results were corroborated by a complementary study showing that neonates, which lack MHCII^hi^ BAMs during the first 3 weeks of life, are highly susceptible to LCMV-induced meningitis.^106^ During infections with the extracellular parasite *Trypanosoma brucei*, BAMs were shown to be important for parasite control in the dura mater during the early stages of the disease.^96^ Similarly, BAMs have been shown to protect against *Streptococcus pneumoniae*, which induces bacterial meningitis.^18^ Interestingly, the authors observed that bacteria can subvert dural BAM defenses by activating nociceptor neurons to release calcitonin gene-related peptide (CGRP). CGRP acts via its receptor RAMP1 to suppress chemokine expression in BAMs.^18^ Preventing CGRP-RAMP1 signaling in BAMs increased bacterial clearance and immune cell infiltration in the dura.^18^ Similarly, BAMs were implicated in immune cell recruitment to the CSF following trypanosomiasis^96^ or intraventricular LPS injection.^97^

The BAM-assisted recruitment of immune cells is an important component of the antimicrobial defense playbook. Whereas after acute inflammation the first wave represents mostly neutrophils, shortly thereafter this is taken over by monocytes and their progeny.^97^ Upon more chronic diseases, such as trypanosome infection, recruited monocyte-derived macrophages can reach extremely high densities in border tissues such as the dura and CP stroma.^96^ Interestingly, to accommodate this increased macrophage differentiation and maintenance, CP epithelial cells start to robustly express CSF1.^97^ The monocyte-derived macrophages that develop during ongoing inflammation maintain distinct transcriptional states as compared with the resident BAMs that were already present prior to infection.^96^^,^^97^ This is likely the result of differentiating monocytes being imprinted by both the tissue niche and the ensuing inflammation.^138^ Monocyte-derived macrophages that develop during inflammation and disease thus may perform complementary functions. It is therefore important not to immediately classify any brain macrophage expressing BAM-related markers as BAMs or BAM derived. Monocytes infiltrating the mouse or human brain parenchyma can differentiate into microglia-like cells that share many BAM-associated markers, including MS4A7, CLEC12A, CD206, and CD163.^78^^,^^79^^,^^80^^,^^81^ In brain vascular pathologies, such as ischemic stroke or intracerebral aneurysms, CD206^+^/CD163^+^ macrophages, which often appear to play a detrimental role, may represent newly recruited monocyte-derived cells.^139^^,^^140^

Monocytes that are recruited during inflammation or shortly after disease resolution may engraft long-term and replace part of the resident BAM compartment.^96^^,^^141^ Post-disease BAMs display an altered transcriptional or epigenetic state, which could affect how these cells respond to secondary insults,^96^^,^^141^ as is also observed in other organs.^142^^,^^143^ It is tempting to speculate that these changes in BAMs may confer an altered susceptibility to neurological disorders, for which there is evidence in patients who have recovered from infectious disease.^144^

Does the antimicrobial defense conferred by BAMs require antigen presentation and T cell activation? In the context of LCMV, loss of MHCII in BAMs did not affect viral load, whereas loss of interferon (IFN) signaling did.^107^ This indicates that BAMs confer innate antimicrobial protection. However, there is evidence that BAMs are involved in antigen presentation during neuroinflammatory disorders. Multiple sclerosis (MS) is an autoimmune disease characterized by the formation of immune-driven demyelinating lesions in the brain and spinal cord.^145^ MS is modeled in mice by immunizing mice against myelin-derived antigens, giving rise to experimental autoimmune encephalomyelitis or EAE.^146^ Studies have shown that in MS and EAE, self-reactive T cells infiltrate the brain parenchyma via perivascular spaces, crossing the BBB primarily around postcapillary venules.^35^^,^^147^ *In vivo* two-photon imaging has shown that, within the perivascular spaces, CD4^+^ T cells get activated by MHCII^+^ perivascular macrophages, which triggers parenchymal T cell invasion across the glia limitans.^148^^,^^149^^,^^150^ However, Mundt et al. suggested that MHCII^+^ BAMs are not required for antigen presentation to encephalitogenic T cells.^151^ This was based on the observation that genetic deletion of MHCII in microglia, BAMs, and dendritic cells (DCs) abrogated EAE development, whereas deletion of MHCII in only microglia and BAMs did not. Although this shows that DCs suffice to locally license encephalitogenic T cells, it does not necessarily mean that MHCII^hi^ BAMs are not involved as there may be redundancy. Due to technical reasons, it was not possible to delete MHCII in all DCs while sparing it in BAMs,^151^ which would have provided definitive information regarding the ability of BAMs to initiate EAE. Conversely, there is clear evidence that BAMs drive CD4^+^ T cell activation during α-synuclein-induced pathology in the context of Parkinson’s disease (PD).^152^ PD is a chronic neurodegenerative disorder characterized by the accumulation of misfolded α-synuclein in intracellular inclusions known as Lewy bodies, which progressively leads to loss of dopaminergic neurons, particularly in the substantia nigra.^153^ In a mouse model of PD, where α-synuclein is overexpressed in dopaminergic neurons of the substantia nigra via viral delivery, CD4^+^ T cell activation is essential for driving pathogenesis.^154^ MHCII deletion in microglia and BAMs suppresses CD4^+^ T cell infiltration and pathology, whereas MHCII deletion in only microglia does not. Additionally, CD4^+^ T cell infiltration is also impaired following the depletion of BAMs.^152^

In conclusion, BAMs are emerging as important border immune gatekeepers in health and disease. Their specific role in antigen presentation and immune cell recruitment, especially in non-communicable diseases, merits further investigation.

## Concluding remarks, perspectives, and recommendations

As outlined in this review, recent work has started to uncover the complex origins, cell states, and functions of macrophages within the brain’s border tissues. This has revealed the essential contributions of BAMs to brain physiology and defense, both during homeostasis and disease. However, these are still early days and many questions remain. The tissue-trophic functions of BAMs may prove to be more extensive. Macrophages can act as important sources of growth factors and signaling molecules.^155^ Given their prime localization within or close to the glymphatic fluid distribution system in the brain, the BAM secretome may regulate tissue growth and function. In fact, BAMs may help to shape the development of the tissues where they reside. As highlighted in this review, emerging evidence suggests that the trophic functions of BAMs that support tissue homeostasis may be primarily associated with the MHCII^low^ subset, whereas MHCII^hi^ BAMs may serve as immune sentinels and effectors. This implies that the two main BAM states reflect functional specialization. We propose that, rather than classifying BAMs based solely on individual markers such as MHCII, CD206, or LYVE1, it may be more informative to define them according to function—for example, as trophic versus sentinel BAMs. However, attributing distinct functions to specific BAM states remains challenging, as there is currently a lack of genetic tools to selectively target only one BAM subset. Although several Cre-drivers have been developed that target BAMs, using endogenous promoters such as *Mrc1*, *Lyve1*, *Pf4*, or *Cd163*, none have been shown to drive subset-specific recombination.^63^^,^^88^^,^^91^^,^^107^ Furthermore, there are currently no genetic tools available that target MHCII^hi^ BAMs without also affecting other antigen-presenting cells.

What are the niche signals and gene-regulatory networks that drive BAM identity? The signals that control BAM activation are likely very different than those for microglia and remain largely unknown. Indeed, a good example is TGF-β, which is essential for microglial development^76^ but seems dispensable for BAMs.^86^ What are the factors driving changes in BAMs across aging, such as the progressive exchange of MHCII^low^ with MHCII^hi^ BAMs? How much does exposure to pathogens and (auto)antigens influence this shift? Equally important is uncovering whether these changes are detrimental to maintaining healthy brain physiology and whether they predispose to disease. Preventing age-related changes in BAMs or finding ways to “rejuvenate” these cells could prove to be therapeutically significant. In this context, Rebejac et al. have shown that depletion of BAMs coupled with anti-integrin blockade favors an expansion of MHCII^low^ BAMs in the dura mater,^107^ whereas Drieu et al. demonstrated that CSF1 treatment in old mice acutely increases matrix metalloproteinase activity in BAMs, leading to enhanced CSF flow.^62^ BAMs are also relevant in the context of neurodegenerative diseases. Human BAMs express GWAS genes, such as *APOE* or *TREM2*, linked to AD and other neurodegenerative or neuroinflammatory disorders.^98^^,^^99^ Until now, most attention has gone to microglia; however, it could be rewarding to uncover the role of BAMs within these disorders. Therapeutic strategies that target BAMs have remained underexplored.

Some considerations may be helpful in the further pursuit of BAM biology. Given that BAMs comprise a heterogeneous population in highly complex tissues, it will be helpful to avoid certain simplifications. As BAMs exist in two main states, it will be important to try to distinguish between these states when considering origin and functions. Moreover, it is currently often considered that all leptomeningeal and perivascular BAMs have an embryonic origin, thereby disregarding MHCII^hi^ BAMs in these locations. Nomenclature is important too. As the dura mater and leptomeninges have highly distinctive features that can clearly affect the origin and functionality of BAMs, it would be better not to use generic terms such as “meningeal BAMs” but to specify the meningeal layer (dural, leptomeningeal BAMs). Related to this, we have previously referred to macrophages in the leptomeninges as subdural BAMs,^5^ but this can also lead to confusion regarding anatomy. Finally, when working with mouse models, careful conclusions need to be made concerning the tissue origin of BAMs when using dissected materials. For example, a mouse brain in which the dorsal and ventral dura is removed can still contain dural tissue within the fissures of the brain. When conclusions need to be made specifically for the leptomeninges, it is better to collect this layer, if possible, or to use leptomeningeal-enriched tissue.^156^ Similarly, it can be challenging to obtain dissected mouse parenchymal tissue that only contains BAMs from perivascular spaces, as the parenchyma is often bordered by meninges or meningeal-like tissues like the tela choroidea, which runs across the ventricles and contains many BAMs as well. A good understanding of the anatomy of the brain and its border tissues is therefore important.

In perspective, BAMs offer a novel key to understanding CNS homeostasis and its communication with the periphery. We look forward to research that will answer existing questions and generate new ones.

## Acknowledgments

This work was supported by FWO grants (G058921N and G042021N), a Collen-Franqui start-up grant and an ERC consolidator grant (101088437 ReplaceMi) to K.M.

## Declaration of interests

The authors declare no competing interests.
